# The Zebrafish Model to Understand Epigenetics in Renal Diseases

**DOI:** 10.3390/ijms22179152

**Published:** 2021-08-25

**Authors:** Nina Sopel, Janina Müller-Deile

**Affiliations:** 1 Department of Medicine 4–Nephrology and Hypertension, Universitätsklinikum Erlangen, 91054 Erlangen, Germany; 2 Department of Nephrology, Friedrich-Alexander-Universität Erlangen-Nürnberg, 91054 Erlangen, Germany

**Keywords:** zebrafish model, epigenetics, renal diseases, microRNAs, histone modifications, DNA methylation

## Abstract

Epigenetic modifications are able to alter gene expression and include DNA methylation, different histone variants, and post-transcriptional modifications (PTMs), such as acetylation or phosphorylation, and through short/long RNAs, respectively. In this review, we focus on current knowledge concerning epigenetic modifications in gene regulation. We describe different forms of epigenetic modifications and explain how epigenetic changes can be detected. The relevance of epigenetics in renal diseases is highlighted with multiple examples and the use of the zebrafish model to study glomerular diseases in general and epigenetics in renal diseases in particular is discussed. We end with an outlook on how to use epigenetic modifications as a therapeutic target for different diseases. Here, the zebrafish model can be employed as a high-throughput screening tool not only to discover epigenetic alterations contributing to disease, but also to test novel substances that change epigenetic signatures in vivo. Therefore, the zebrafish model harbors the opportunity to find novel pathogenic pathways allowing a pre-selection of potential targets and compounds to be tested for renal diseases.

## 1. Epigenetic Modifications

The term epigenetics describes patterns of modifications of the DNA, resulting in differential gene expression, while the genomic sequence is not altered.

**Figure 1:**
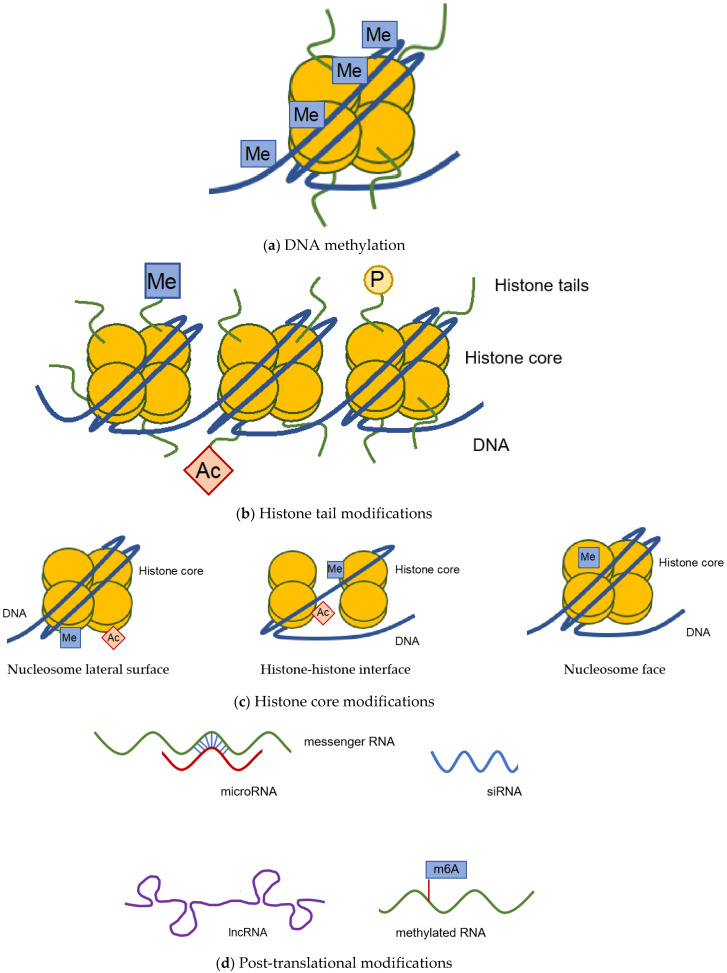
Schematic depiction of epigenetic modifications. Epigenetic modifications describe a set of different chemical alterations to either DNA, histones, or RNA that influence gene expression, without changing the genomic sequence. DNA is methylated (**a**) and the status of this methylation, as in hyper- or hypomethylation, has an impact on gene expression [1]. Furthermore, histones can be chemically modified on either their tails (**b**) or on the histone core itself (**c**). For histones, several modifications have been described, in addition to methylation, acetylation, phosphorylation, and SUMOylation have also been found. Histone cores can be modified on their lateral surface, the histone–histone interface, or the nucleosome face [2]. (**d**) Post-translational modifications of RNA are mediated by microRNAs, small RNAs, such as siRNAs, or long non-coding RNAs. Methylation of mRNA molecules has also been described [3,4]. Figures adapted from [2,5].

In general, there are three different modes of epigenetic modifications—DNA methylation, histone modifications, and posttranscriptional modifications through short/long RNAs (Figure 1).

### 1.1. DNA Methylation

By adding methyl groups to the DNA, transcription of the respective DNA segment is altered (Figure 1a). While most invertebrates display a mosaic pattern of DNA methylation throughout their genome, vertebrae genomes are predominantly methylated at CpG dinucleotides. Due to the high mutagenic potential of methylcytosine, which can deaminate spontaneously and cause cytosine to thymine transitions, the genome of vertebrates is in general CpG-poor. Yet, there are so-called CpG islands, which have a content of more than 50% of guanines and cytosines and are longer than 200 bp, that are usually unmethylated and represent key regulatory units. These are found predominantly in promoter regions of developmental and housekeeping genes. High DNA methylation is found in heterochromatin and repeat elements, while gene-distal regulatory elements, such as enhancers, which have a moderate CpG content, are differentially methylated depending on the type of cell. DNA methylation is involved in several physiologic processes, such as genomic imprinting, inactivation of the X chromosome, and aging and has been shown to be essential for development [1,6,7,8].

Methyl groups can be added to either adenine or cytosine, however, in mammals the most common and most studied form of methylation is that in CpG dinucleotides. Recently also non-CpG methylation has been described e.g., in embryonic stem cells, hematopoietic progenitor cells, or brain development [9,10,11].

DNA is methylated in defined regions spanning several 100 kb via DNA-methyltransferases (DNMTs). DNMT3A, DNMT3B, and DNMT3C are de novo methylation enzymes, which contain a highly conserved MTase domain and two chromatin reading domains. DNMT3L is catalytically inactive, but stimulates DNMT3A and B activity and interacts with those enzymes in the germline. Symmetrical CpG methylation is maintained in replication of DNA, this is facilitated by DNMT1 together with an E3 ubiquitin-protein ligase named UHRF1. Ten-eleven translocation (TET) enzymes are able to actively demethylate DNA and it has been shown that both DNMTs and TETs can be co-expressed, causing turnover of DNA methylation, a feature that has recently been described in the differentiation of pluripotent stem cells [7,8].

### 1.2. Histone Modification

In contrast to DNA methylation, modification of histones is more variable, as in addition to methylation, acetylation, biotinylation, SUMOylation, phosphorylation, and other chemical modifications can occur and the changes of these modifications are reversible, which means that gene transcription is repressed or enhanced. Enzymes involved in these processes include e.g., histone-deacetylases (HDACs), histone-acetyltransferases (HATs), and histone-methyltransferases (HMTs).

Histone modifications can alter the structure of the chromatin, making it more or less accessible for transcription [12].

Each histone represents an octamer of core histones, with two H2A–H2B dimers and one H3–H4 tetramer. DNA is wrapped around the core histone in 1.65 turns (~146 bp), forming a higher order structure, which is stabilized by H1, the linker histone.

Histone proteins are highly conserved and post-translational modifications occur on both the N-terminal tails and on globular domains. Histone tail modifications (Figure 1b), such as methylation at lysine 4 of histone H3 (H3K4), have been described to be involved in DNA repair, replication, and transcription, as well as indicating enhancer regions. However, these modifications do not affect chromatin structure itself, but are believed to either recruit or ward off proteins that bind specifically to post-translational modifications, which then facilitate the observed biological effects. Recently, histone core modifications (Figure 1c) on the histone lateral surface (e.g., H3 K122), histone–histone interface (e.g., H4 K91) and the solute-accessible face (e.g., H4 K59) have been identified. These are proposed to directly regulate the accessibility of nucleosomal DNA to regulatory factors and thereby contribute to changes in chromatin-dependent processes, such as DNA damage repair, transcription, and chromatin assembly [2,13,14].

### 1.3. Post-Transcriptional Modification

Gene expression can also be modified posttranscriptionally by non-coding RNAs (Figure 1d). These include microRNAs (miRNAs), short RNAs, and long RNAs. Short noncoding RNAs can be divided into three classes, namely microRNAs (miRNA), small interfering RNAs (siRNA), and piwi-interacting RNAs (piRNA). Long non-coding RNAs (lncRNAs) modify gene expression on the chromatin level [3]. Short RNAs and lncRNAs interact with each other with reciprocal consequences for their fates and functions. piRNAs induce epigenetic and post-transcriptional silencing of transposons [15].

The principle of RNA interference mediated by siRNAs and miRNAs is based on three steps. First, long double-stranded RNAs are enzymatically processed into smaller fragments by RNase III enzymes (Dicer and Drosha). These double-stranded fragments are then separated, and the leading stand is loaded to the RNA-induced transcriptional silencing complex (RISC). The RISC is then able to find its targeted RNA and either inhibit or degrade it, whereas the small RNAs give the specificity and the Argonaut protein within the RISC conveys repression of translation. For siRNAs, Argonaut proteins cleave target RNA in several cycles, while the guide strand is bound. In the case of miRNAs, Argonaut proteins inhibit translation by staying associated with the target RNA or induce degradation of the poly-A tail of the target RNA, resulting in RNA degradation [16,17].

Furthermore, similar to DNA, RNA molecules can also be chemically modified. Here, N6-methyladenosin (m6A) was shown to be the most abundant from of modification in eukaryotic RNA. It is involved on many levels in mRNA processing, e.g., in mRNA degradation, but also in promoting mRNA translation and export from the nucleus. It has also been linked to human disease, e.g., neuronal disorders, viral infection, and inflammation [4,18]. In addition to mRNAs, functional RNAs, namely transfer RNAs (tRNAs) and ribosomal RNAs (rRNAs) are also chemically modified for stabilization and proper biological functions. While the exact pathological mechanisms are still elusive, changes in these modifications or their mediators have already been linked to human disease, e.g., dyskeratosis congenita [4,18].

## 2. Methods to Detect Epigenetic Changes

Different methods have been described to detect epigenetic changes. Some are specific for the type of modification, as described below, others can be used to detect e.g., methylation in general. Furthermore, indirect methods are available and include the analysis of modulating enzymes that are involved in epigenetic changes, such as methyl CpG binding proteins, HDACs or DNMTs [19].

### 2.1. Detection of DNA Methylation

The most common method to detect DNA methylation is bisulfite conversion. Here, DNA samples are treated with sodium bisulfite, resulting in the deamination of unmethylated cytosine to uracil, while methylated cytosine residues are protected from deamination. The downstream analyses to distinguish between cytosine and uracil are manifold. Different sequencing approaches, such as next-generation sequencing (NGS) or pyrosequencing give single-site resolution of the methylation status and give a quantitative result. The use of methylation-specific PCR gives no single-site resolution and is only semi-quantitative. Another method to analyze bisulfite-converted DNA is mass spectrometry (MS), where mass shifts can be detected and used to identify methylation sites and other posttranscriptional modifications, such as histone modifications [2]. More specifically, matrix-assisted laser desorption/ionization—time of flight (MALDI-TOF) analysis, where the sample is ionized by transferring energy from a surrounding matrix after a laser pulse and these ions are then accelerated in a mass spectrometer to measure time of flight—is used to detect epigenetic modifications [20,21]. For example in renal cell carcinoma, it has been shown that MALDI-TOF can be used to reproducibly quantify the methylation status of individual DNA bases [2,19,22,23]. In addition, bisulfite-converted DNA can be subjected to high resolution melt analysis. After bisulfite conversion and PCR amplification, the PCR product is subjected to decreasing temperatures and the melt temperature and melt curve profile are obtained [24]. In a different approach, methylated DNA immunoprecipitation (MeDIP) enables examination of genome-wide changes in DNA methylation patterns. γH2AX immunostaining is a marker of DNA double-strand breaks [25], while 5-methyl cytosine immunostaining is a marker of DNA methylation [26]. Furthermore, DNA enzyme digest can be used for analysis of DNA methylation status, where digestion of specific DNA target sequences by DNA endonucleases, which do not cut methylated DNA, generates fragments of different lengths, which are then sequenced to determine the extent of methylation [24].

### 2.2. Analyzing Histone Modifications

Traditionally, histone modifications have been identified by using antibody-based assays, such as anti-histone antibodies in Western blot, ELISA, or identification of modified histone-interacting proteins by immunoprecipitation. Furthermore, the genomic locations of histone marks are analyzed by chromatin immunoprecipitation (ChIP). With ChIP analysis, interactions between DNA and protein are examined; as in a first step DNA and protein are cross-linked by formaldehyde treatment, followed by fragmentation, subsequent immunoprecipitation, and finally analysis with either PCR, a sequencing approach, such as NGS, or microarray strategies [19,27].

Recently, mass spectrometry has frequently been used to detect histone modifications, where different variations of MS are applied, such as LC-MALDI-TOF and LC-MS/MS [2,28,29].

### 2.3. Post-Transcriptional Modifications

Traditional methods for detection of miRNAs include Northern blotting, quantitative reverse transcription polymerase chain reaction (qRT-PCR), next-generation sequencing, and microarray-based hybridization [30,31]. Furthermore, mass spectrometry, bisulfite sequencing or antibody-based approaches are widely used for analysis of post-transcriptional modifications [32,33].

However, it is important to keep in mind that with total RNA isolation all RNA types in the cell are isolated and further purification steps are needed to obtain e.g., messenger or ribosomal RNA. Methods described for the detection of RNA modifications include radioisotope incorporation assays, thin-layer chromatography, and antibody-based sequencing. Furthermore, there are methods to detect modifications to the sugar–phosphate backbone of the RNA, e.g., subjecting RNA to alkali hydrolysis, which is then ligated to an adaptor prior to reverse transcription. RNA that is methylated at the 2′-O-ribose position is resistant to alkali hydrolysis at neighboring nucleotides and therefore a gap is revealed when this RNA is sequenced [34].

## 3. Epigenetics in Renal Diseases

Epigenetic modifications have been described to be involved in the pathophysiology of many different diseases and first studies in glomerular diseases have been conducted.

### 3.1. DNA Methylation

In patients with IgA nephropathy (IgAN), Hayashi et al. found a correlation between glomerular DNA double-strand breaks and DNA methylation, an association of podocyte DNA double-strand breaks with podocytopathic features, and an association of glomerular γH2AX with the slope of eGFR decline [26].

Furthermore, sirtuin 1 (Sirt1) is a NAD+-regulated deacetylase and has been described to have protective effects in diabetes. Tubular-specific overexpression of SIRT1 induced hypermethylation of the *Cldn1* gene leading to downregulation of the tight junction protein Claudin-1 in podocytes, which protected against albuminuria and suggested a cross-talk between proximal tubules and podocytes [35].

In addition, the recently identified biomarker Klotho, which is attributed to have reno-protective characteristics, e.g., by direct binding to the receptor for transforming growth factor beta (TGFβ-R), has been shown to be hypermethylated at its promoter in acute and chronic kidney disease (CKD). A possible treatment with the rhubarb-derived compound rhein was suggested to alter the aberrant promoter methylation status [36].

### 3.2. Histone Modifications

Alteration of histone H3K4 methylation in glomerular podocytes was associated with proteinuria in patients with membranous nephropathy [37]. The role of histone epigenetics in development and progression of diabetic nephropathy was also studied in db/db mice. Here, advanced diabetic nephropathy was correlated with increased renal H3K9 and H3K23 acetylation, H3K4 dimethylation, and H3 phosphorylation [38]. Furthermore, in diabetic rats, enhanced histone acetylation on genes of the natriuretic peptide system (NPS), namely *Nppa*, *Nppb*, and *Nme*, was observed. These genes were previously associated with the pathogenesis of both cardiomyopathy and nephropathy. The authors suggest that hyperglycemia and other metabolic stressors activate pathological signaling cascades, which in turn induce upregulation of e.g., histone acetyl transferases, leading to global histone acetylation in the kidneys and hearts of diabetic patients [39]. In line with this suggestion, Jia et al. proposed a dysregulation of methylases, demethylases, and miRNAs that contribute to glomerular damage in diabetic nephropathy. They showed that TGF-β1, which is a key promoter of fibrosis in glomerular diseases, increased the expression of histone demethylases JMJD3 and UTX and downregulated the H3K27me3 methyltransferase enhancer of Zeste homolog 2 (Ezh2) via miRNA-101b in mesangial cells under high-glucose treatment [40].

### 3.3. Post-Transcriptional Modifications

While data on other epigenetic changes in glomerular disease is slowly expanding, alterations in post-transcriptional modifications have already been reported abundantly. Here, we can only mention some of these findings.

Aberrant O-glycosylation in the hinge region of IgA1 characterizes IgAN. Reduced expression of the enzyme core 1, β1,3-galactosyltransferase 1 (C1GALT1), was suggested to be responsible for this phenomenon. miRNA-148b, miRNA-374b, and let-7b have been shown to regulate galactosylation of IgA1 in peripheral blood mononuclear cells (PBMCs). miRNA-148b and let-7b inhibit the expression of the C1GALT1 and GALNT2 enzymes at the mRNA and protein expression levels, whereas miRNA-148b correlated positively with the concentration of poorly galactosylated IgA1 [41]. In a different study, serum levels of let-7b and miRNA-148b, that regulate the O-glycosylation process of IgA1, appeared to be non-invasive markers to predict the probability of having IgAN [42]. Plasma miRNA-148a-3p, miRNA-150-5p, miRNA-20a-5p, and miRNA-425-3p were shown to be significantly upregulated in patients with IgAN compared to controls [43]. Furthermore, miRNA148a-3p correlated positively with eGFR. MiRNA expression in urinary sediments from patients with IgAN was also found to be altered. Urinary expression of miRNA-3613-3p was downregulated and both miRNA-3613-3p and miRNA-4668-5p correlated with disease severity [44]. Another study found significant differences in miRNA-150, miRNA-204, miRNA-431, and miRNA-555 expression between IgAN and healthy controls in the urine sediment [45].

Patients with focal segmental glomerulosclerosis (FSGS) were shown to have elevated urinary miRNA-3d and miRNA-10a in the urine compared to healthy controls [46]. Recently, we found miRNA-378a-3p upregulated in urine and kidney biopsies from patients with membranous glomerulonephritis (MGN) [47].

Peripheral blood from patients with diabetes and the kidneys of animals with type 1 or 2 diabetes expressed lower miRNA-25 than nondiabetic controls. Systemic administration of a miRNA-25 antagomir repressed glomerular fibrosis and reduced high blood pressure in db/db mice, indicating a potential novel therapeutic target for diabetic nephropathy [48]. TGF-β upregulates different miRNAs including miRNA-21 and accelerates podocyte loss and glomerulosclerosis. miRNA-21-deficient TGF-β1-transgenic mice or streptozotocin-induced diabetic mice showed increased proteinuria, podocyte depletion, and glomerular extracellular matrix deposition. In patients with diabetic nephropathy, albumin-to-creatinine ratio was positively associated with miRNA-21 expression in glomerular fractions. The authors suggested miRNA-21 as a feedback inhibitor of TGF-β signaling and functions [49].

In addition to miRNAs, lncRNAs have also been described in kidney disease [50]. For example, lncRNA LOC105374325 was upregulated in podocytes of FSGS patients and induced podocyte apoptosis by serving as a sponge for miRNA-34c and miRNA-196a/b [51]. The lncRNAs, Xist, and NEAT1 were significantly upregulated in tubular epithelial and glomerular cells in a mouse model for membranous glomerulonephritis. Furthermore, urinary Xist correlated with disease severity and was suggested as a potential noninvasive marker for membranous glomerulonephritis [52]. Xist seems to have proapoptotic effects on podocytes through sequestration of miRNA-217 and consecutive upregulation of Toll-like receptor 4 (TLR4) [53]. Elevated lncRNA RP11-2B6.2 was found in kidney biopsies from patients with lupus nephritis. This lncRNA positively correlated with disease activity. LncRNA RP11-2B6.2 was further identified as a positive regulator of the IFN-I pathway through epigenetic inhibition of SOCS1 [54].

## 4. Using the Zebrafish Model to Study Epigenetics in Renal Diseases

In recent years, the zebrafish (*Danio rerio*) model has been used to study glomerular function and disease [47,55,56,57,58]. The zebrafish larvae’s pronephros, which is composed of two bilateral pronephric ducts linked with fused glomeruli in the midline of the larvae, is very similar to the human metanephros [59,60]. The pronephros tubular epithelium is composed of two proximal convoluted tubules, two proximal straight tubules, two distal early and distal late tubule segments, and a pronephric duct [61]. The main difference between the pronephros of the zebrafish and the mammalian partner is that the pronephros does not have a thin limb segment between the proximal straight tubule and the thick ascending limb. The glomerulus of the pronephros contains podocytes, glomerular basement membrane fenestrated endothelial cells, and mesangial cells [56]. Glomerular filtration begins as early as 48 h post-fertilization (hpf) and a fully functioning pronephros of zebrafish larvae is fully developed within 72 hpf [62,63].

At least 70% of zebrafish proteins have a human orthologue [64]. Furthermore, the zebrafish is very amenable to genetic manipulations though microinjections of morpholinos, DNAs, RNAs, and microRNAs [47,56,57,58,65,66,67,68,69].

Given its high genetic and renal similarity to humans the zebrafish has been used to study different renal diseases such as FSGS [70,71,72,73], polycystic kidney diseases [74,75,76], diabetic nephropathy [73,77], and renal cancer [78].

However, these manipulations can have effects on the developing embryo and observed phenotypes might not be specific to the tissue or organ of interest. Furthermore, injection of these molecules often has off-target effects, which are sometimes difficult to identify.

For more specificity, genetic editing techniques such as transcription activator-like effector nucleases (TALENs), zinc finger nucleases, and clustered regularly interspaced short palindromic repeat (CRISPR)-Cas9 can also be performed in the zebrafish [79,80,81].

The zebrafish has already served as a model to investigate epigenetic changes in hearing loss [31], development [82], and cancer [78,83,84] and in propagating secondary complications observed in diabetes mellitus [85]. However, though the zebrafish model has been used abundantly in kidney research, so far only few studies have focused on the epigenetic contribution to renal diseases in this versatile in vivo model [47,57,58,86].

### 4.1. DNA Methylation

It has been observed that loss of DNA methylation in developing zebrafish embryos upregulates LTR transposons, which were shown to be highly methylated in control larvae. This hints a direct contribution of DNA methylation in suppression of this type of transposable element [87]. Furthermore, dysregulated expression of stem cell transcription factor POU5F1 is part of a larger pattern of gene expression changes in renal cell cancer that may be induced by HIF-dependent reactivation of dormant promoters embedded within endogenous retroviral LTRs [88].

### 4.2. Histone Modifications

Ito et al. have demonstrated that in podocytes Wolf–Hirschhorn syndrome candidate 1-like 1 long form (WHSC1L1-L) acts as a histone methyltransferase and suppresses nephrin gene expression by binding to its promoter, probably by reducing H3K4 trimethylation. This alteration of nephrin expression might be involved in both acquired and congenital nephrotic syndrome [89]. The zebrafish model was also used to generate a bioinformatic pipeline in dissecting functions of kidney-disease-associated variants based on cell-type-specific epigenome including transcription-centered 3D chromatin organization and histone modifications [83].

### 4.3. Post-Transcriptional Modifications

Data on post-transcriptional regulation is more abundant concerning renal disease. siRNA-mediated gene-silencing techniques have been used to examine e.g., heart regeneration in zebrafish. In the study of Xiao et al., nanoparticles, which encapsulated a siRNA specific to *Aldh1a2* (aldehyde dehydrogenase 1 family, member A2), were injected into zebrafish hearts after resection of the apex of the ventricle and knock-down of the target gene was observed [90]. This method provides an alternative approach for determining gene functions in zebrafish and might be extended to other organs such as the kidney. Recently, we have shown that e.g., microRNA-26a-5p is increased in urine of preeclamptic patients, compared to healthy controls, and that miRNA-26a-5p overexpression in zebrafish leads to a phenotype comparable to that of preeclampsia, including proteinuria, edema, and podocyte foot-process effacement [58]. Among others, vascular endothelial growth factor A (VEGF-A) is an important target of miRNA-26a-5p and reduced VEGF-A levels are a hallmark of preeclampsia. We not only showed that overexpression of miRNA-26a-5p in zebrafish reduces vegf-A but also that injection of human VEGF-A protein was able to partially rescue the miRNA-26a-5p-induced phenotype [58].

We also observed that miRNA-143-3p decreased syndecan 3 and 4, as well as versican mRNA after overexpression in zebrafish larvae. These components of the glycocalyx that are produced by podocytes and glomerular endothelial cells have an important role in a well-functioning glomerular filtration barrier. Overexpression of miRNA-143-3p in zebrafish, by injection of a miRNA-143-3p, mimicked the one to four cell stage causing edema, podocyte effacement, loss of plasma proteins, and endothelial damage [57].

MiRNA-378a-3p targets podocyte nephronectin (NPNT), an extracellular matrix protein in the GBM. miRNA-378a-3p mimic injection, as well as npnt knockdown by a morpholino caused a similar phenotype consisting of edema, proteinuria, podocyte effacement, and widening of the glomerular basement membrane in zebrafish. Murine Npnt constructs containing a mutated 3′UTR region prevented the phenotype caused by miRNA-378a-3p mimic injection and indicated that the miRNA-induced changes in npnt caused the pathological findings. Biopsies from patients with FSGS and MGN showed increased miRNA-378a-3p expression and reduced glomerular levels of NPNT suggesting miRNA-378a-3p-mediated suppression of NPNT as a novel mechanism for proteinuria in glomerular diseases [47].

Epigenetic gene regulation and transcription-factor-mediated regulation share similarities, as usually both are involved in the regulation of gene expression. There is a tight interaction between epigenetic modifications and transcription factors as transcription factors can induce epigenetic changes and promoters of transcription factors have been found themselves to be modified by epigenetic regulators [91]. Several studies using the zebrafish model have been conducted to analyze transcription factor contribution to renal disease. For example, activation of P-TEFb by cAMP-PKA signaling was studied in autosomal dominant polycystic kidney disease (ADPKD) [92]. Furthermore, loss of vhl in the zebrafish pronephros recapitulates early stages of human clear cell renal cell carcinoma by stabilization of HIF1a and HIF2a, which up-regulates specific target genes involved in cell proliferation, angiogenesis, and erythropoiesis [92]. The transcription factor Dach1 was found to be important for podocyte differentiation and proper kidney function [93]. Finally, mutation in microphthalmia-associated transcription factor/melanogenesis-associated transcription factor (mitf) caused a significantly higher susceptibility for the disruption of the glomerular filtration barrier following puromycin treatment in zebrafish [86].

Table 1 summarizes current studies using the zebrafish model to study epigenetics and transcription factors in renal disease.

## 5. Epigenetic Modifications as a Therapeutic Target for Renal Diseases

Unlike gene therapy, epigenetic therapies are reversible and drug-able for targeted approaches. Several drugs targeting epigenetic regulators are in clinical development or use, however, they are so far mostly used for malignant diseases, such as acute myeloid leukemia (AML) or myelodysplastic syndrome (MDS) [94].

### 5.1. DNA Methylation

Drugs that alter the methylation status of DNA by either activating or inhibiting DNA methylation, such as hydralazine or 5′azacytidine, respectively, are mainly used for the treatment of non-solid tumors. Although there have been reports that these agents negatively affect kidney function, they have now also approved for use in patients with compromised kidney function, however close monitoring is advised [94,95]. Class I histone deacetylase inhibitors, such as valproic acid, have been used in experimental kidney injury and have shown promising results [94,96]. Another DNA-demethylating drug is hydralazine. While in higher doses it is used as an antihypertensive drug, it has been shown to demethylate DNA effectively without an effect on blood pressure in lower doses. Preclinical results have suggested that low-dose administration of hydralazine during gestation to female mice on a high fat diet has a preventive effect on CKD development in their offspring [97].

In addition, the positive effect of rhein on klotho-promoter methylation in an adenine-mediated model of CKD further promotes the idea of using drugs to alter the DNA methylation status for treating kidney patients [36].

### 5.2. Histone Modifications

Different agents to alter histone modifications have been tested in phase I or phase II trials for non-solid tumors, but no data on kidney disease have been generated [94]. However, in previous studies it has been suggested that upregulation of histone H3K4 trimethylation is involved in pathology of MGN and podocyte dysfunction. Here, podocyte histone H3K4 me3 was negatively correlated with synaptopodin and positively correlated with proteinuria. Therefore, reducing this epigenetic signature might be effective in reducing the renal damage observed in MGN [37].

Furthermore, Malek et al. suggest that histone acetylation inhibitors might be novel candidates for treatment of diabetic nephropathy and diabetic cardiomyopathy by increasing histone acetylation in promoter regions of genes encoding for peptides of the natriuretic peptide system [39].

Interference with histone modification readers e.g., by inhibition of bromodomain and extra-terminal proteins (BET) by apabetalone is currently in phase III trials for atherosclerosis [94]. Here, kidney function is a secondary endpoint of the study.

Interestingly, in recent studies, SGLT2 inhibitors have been shown to have multiple reno-protective effects by increasing the circulating and tissue levels of β-hydroxybutyrate, a molecule that generates a specific histone modification [98].

### 5.3. Post-Transcriptional Modifications

Antisense techniques that employ synthetic oligonucleotides have been used to interfere with miRNA function and might have potential as a novel therapeutic strategy. Anti-miRNA oligonucleotides (AMOs) are synthetic reverse complements of miRNAs that tightly bind and inactivate the miRNA. AMOs bind with high affinity to the miRNA ‘seed region’. The use of chemically engineered antisense oligonucleotides targeting specific miRNAs is challenged by circulating RNases and pH-dependent degradation. A variety of chemical modifications can be applied to improve the performance and potency of AMOs. Chemical modifications are mainly introduced in the sugar ring and/or in the backbone of the oligonucleotide structure. The most popular alterations applied to miRNAs studies involve modification at the 2′ carbon of the ribose (2′-F-RNA, 2′-O-methyl- or 2′-O-methoxyethyloligonucleotides). Other chemical modifications include phosphorothioate-containing oligonucleotides [99], locked nucleic acids (LNA), oligonucleotides [100], and peptide nucleic acids [101]. Backbone modifications with phosphodiester bonds are natural internucleotide linkage strategies [101].

The most prominent examples for miRNA therapy in glomerular diseases is in Alport disease. MiRNA-21 expression was significantly elevated in kidney specimens from patients with Alport syndrome and correlated with proteinuria, serum creatinine, and severity of kidney pathology. Administration of anti-miRNA-21 to Alport mice slowed decline in kidney function and improved survival [102]. On the other hand, inhibition of miRNA-21 was protective against TGF-β-induced fibrogenesis and inflammation in different renal cell types [103]. In a phase-1, open-label, multi-center study, safety, pharmacodynamics, and pharmacokinetics of the anti-miRNA-21 agent called RG-012 were investigated in patients with Alport syndrome (NCT03373786). The study consisted of two parts (Part A and Part B). During Part A, half of the participants received a single dose of RG-012 and the other half received four doses of RG-012. All subjects underwent two renal biopsies, one before and one after receiving RG-012, to assess the effect of RG-012 on the kidney. Even though the study has been completed, results have not yet been published. Another phase 2, randomized, double-blind, placebo-controlled study to evaluate the safety, efficacy, pharmacodynamics, and pharmacokinetics of anti-miRNA-21 agent Lademirsen (SAR339375) for subcutaneous injection administered every week in patients with Alport syndrome is on its way (NCT02855268).

As most dominant forms of FSGS are caused by gain-of-function mutations these forms of FSGS might also be attractive for epigenetic therapy by silencing gene expression. For example, mutations in TRPC6 appear to be gain-of-function, leading to increased channel activity [104,105].

## 6. Conclusions and Outlook

In summary, alterations of epigenetic modifications have been shown to contribute to different human diseases including renal diseases. To date, the use of drugs that alter the epigenetic state of genes and histones has been utilized mostly in the treatment of non-solid tumors, but preclinical data also suggest broader fields of application in renal disease.

**Figure 2:**
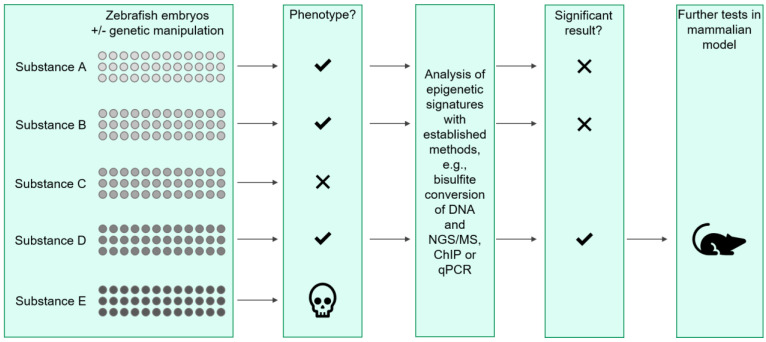
Schematic workflow for high throughput screening of different compounds in the zebrafish model. In a first step, zebrafish embryos with or without genetic manipulation via e.g., morpholino injection, are incubated in the presence of different substances (substances A to E in this example). The embryos are monitored to detect toxicity, developmental delay, and changes in phenotype. If the embryos develop phenotypically normal, fish are sacrificed and analyses of epigenetic changes are conducted, e.g., with bisulfite conversion of DNA with subsequent next-generation sequencing (NGS) or mass spectrometry (MS). Further evaluations include chromatin immune precipitation (ChIP) and quantitative PCR (qPCR). If significant results regarding the effect of the specific substance are obtained, the substance is tested further in a mammalian, e.g., murine, model.

Here, the use of the zebrafish model can be employed as a high-throughput screening tool not only to detect epigenetic alterations contributing to disease but also to test novel substances that cause changes in the epigenetic signatures in vivo. An exemplary workflow of how these experiments could be performed is depicted in Figure 2.

Even though results have to be confirmed in other animal models, the zebrafish model gives an opportunity to make a pre-selection on potential targets and compounds with data on toxicity, tissue damage, and bioavailability in a vertebrae model.

## Figures and Tables

**Table 1:** Studies using the zebrafish model to study epigenetics and transcription factors in renal disease.

Study	Renal Disease	Epigenetic Mechanisms/Transcription Factors Involved	Results
“Overexpression of TGF-β Inducible microRNA-143 in Zebrafish Leads to Impairment of the Glomerular Filtration Barrier by Targeting Proteoglycans”; Müller-Deile et al. [57]	FSGS (focal segmental glomerulosclerosis)	Downregulation of versian and syndecan by miR-143-3p	Proteinuria, edema, and podocyte effacement
“Podocytes regulate the glomerular basement membrane protein nephronectin by means of miR-378a-3p in glomerular diseases”; Müller-Deile et al. [47]	Membranous glomerulonephritis	Downregulation of nephronectin by miR-367a-3p	Proteinuria, edema, podocyte effacement, and disrupted glomerular basement membrane
“Overexpression of preeclampsia-induced microRNA-26a-5p leads to proteinuria in zebrafish”; Müller-Deile et al. [58]	Preeclampsia	Downregulation of vascular endothelial growth factor A (VEGF-A) by miR-26a-5p	Proteinuria, edema, and glomerular endotheliosis
“Chromatin architecture reveals cell-type-specific target genes for kidney disease risk variants”; Duan et al. [83]	Risk variants for renal tumor and chronic kidney disease	Histone modifications of risk variants	Renal tumor and chronic kidney disease
“Activation of P-TEFb by cAMP-PKA signaling in autosomal dominant polycystic kidney disease”; Sun et al. [92]	ADPKD (autosomal dominant polycystic kidney disease)	cAMP-PKA signaling disrupts the inactive P-TEFb/HEXIM1/7SK snRNP complex	Cystogenesis
“Wolf–Hirschhorn syndrome candidate 1-like 1 epigenetically regulates nephrin gene expression”; Ito et al. [89]	Nephrotic syndrome	Wolf–-Hirschhorn syndrome candidate 1-like (WHSC1L1-L) acts as a histone methyltransferase and regulates nephrin gene expression	Reduction of nephrin mRNA
“Loss of vhl in the zebrafish pronephros recapitulates early stages of human clear cell renal cell carcinoma”; Noonan et al. [78]	Clear cell renal cell carcinoma	von Hippel-Lindau (vhl) inactivation leads to > Stabilization of hypoxia-inducible factors 1a and 2a (HIF1a and HIF2a) > Upregulation of specific target genes involved in cell proliferation, angiogenesis and erythropoiesis	Increased tubule diameter, disorganized cilia, cytoplasmic lipid vesicles, glycogen accumulation, aberrant cell proliferation, and abnormal apoptosis
“The transcription factor Dach1 is essential for podocyte function”; Endlich et al. [93]	Podocyte differentiation and proper kidney function	Transcription factor Dach1	Downregulation of nephrin, edema, and leakage of the filtration barrier
“Mutation of microphthalmia-associated transcription factor (mitf) in zebrafish sensitizes for glomerulopathy”; Müller-Deile et al. [86]	Glomerulopathy	Mutation in microphthalmia-associated transcription factor (mitf)	Increased susceptibility to edema, ptoteinuria, and podocyte effacement after puromycin treatment

## Data Availability

Not applicable.
